# Insights into human muscle biology from human primary skeletal muscle cell culture

**DOI:** 10.1007/s10974-025-09696-w

**Published:** 2025-05-10

**Authors:** Thomas Francis, Casper Soendenbroe, Norman R. Lazarus, Abigail L. Mackey, Stephen D. R. Harridge

**Affiliations:** 1 Centre for Human & Applied Physiological Sciences, Basic & Medical Biosciences, Faculty of Life Science & Medicine, King’s College London, London, UK; 2 Institute of Sports Medicine Copenhagen, Department of Orthopaedic Surgery, Copenhagen University Hospital-Bispebjerg and Frederiksberg, Copenhagen, Denmark; 3 Center for Healthy Aging, Department of Clinical Medicine, Faculty of Health and Medical Sciences, University of Copenhagen, Copenhagen, Denmark

**Keywords:** Human, Skeletal muscle, Satellite cells, Myoblasts, Fibroblasts, Cell culture

## Abstract

This review arises from the symposium held in honour of Prof Jenny Morgan at UCL in 2024 and the authors would like to acknowledge the outstanding contribution that Prof Morgan has made to the field of translational muscle cell biology. Prof Morgan published a review article in 2010 entitled: Are human and mice satellite cells really the same? In which the authors highlighted differences between species which are still pertinent to skeletal muscle cell culture studies today. To our knowledge there are no comprehensive reviews which outline the considerable work that has been undertaken using human primary skeletal muscle origin cells as the main model system. This review highlights the multitude of muscle biology that has been investigated using human primary cells, as well as discussing the advantages and disadvantages over other cell models. We also discuss future directions for primary cell culture models utilising the latest technologies in cell type specificity and culture systems.

## Introduction

Part of Prof Morgan’s vast contribution to the field of skeletal muscle biology in health and disease was highlighting that although there are many similarities in muscle physiology across species there are also fundamental differences (Boldrin et al. 2010). Protein expression, cellular proliferative capacity, response to serum, and adherence to different substrates, all differentiate human myogenic cells from those of other species such as mice (Boldrin et al. 2010). Furthermore, there is heterogeneity in samples obtained from different people, producing subtle variations in cellular function, yet how these variations impinge on whole organism function is not known. Similarly, heterogeneity in single, ostensibly homogeneous cell types also occurs. It is also not completely clear which functions a cell may lose as a result of being removed from an in vivo environment. Keeping the foregoing in mind, the impact of these complexities will be examined in more detail as they relate to the muscle cell types under discussion.

Previous reviews have focused on myogenic cell culture in general, combining data from multiple model organisms (Allen et al. 2023; Sharples and Stewart 2011). In this review we focus on research that uses data from human primary skeletal muscle derived cells as the main experimental model and not as an adjunct supporting animal studies. We discuss the advantages, challenges, insights and limitations of studies on human mononucleated muscle derived cells, primarily focusing on the behaviour of myoblasts and fibroblasts in vitro. Myoblasts are the proliferating progeny of muscle stem cells, the satellite cells, which go on to differentiate into myotubes and are characterised by their expression of desmin, the intermediate-filament protein now considered to be the classical marker of mammalian myogenic precursor cells (Baroffio et al. 1995; Kaufman and Foster 1988). By contrast skeletal muscle origin fibroblasts are desmin negative and express classical fibroblastic markers such as collagen VI, vimentin, fibronectin and TE7 (Agley et al. 2013; Stewart et al. 2003).

## Historical muscle cell culture

Evidence for the investigation of muscle regeneration dates back to the late 19th Century followed by the first photographic evidence of cultured muscle origin cells from chickens occurring as early as 1917 (Scharner and Zammit 2011). The proceeding years encapsulated the investigation of many features of muscle regeneration using primary cells obtained from all manner of species including bat, mouse, rat, guineapig and rabbit (Engquist and Zammit 2021). These works predate quite substantially the seminal work of Mauro and Katz separately in 1960 which identified satellite cells in a niche between the basal lamina and sarcolemma of individual muscle cells and which are now known to be the source of muscle progenitor cells (Katz 1961; Mauro 1961).

Since their identification, much work has established these Pax 7 positive satellite cells as the muscle stem cells now known to be essential for repair and regeneration through proliferation and donation of their progeny nuclei to facilitate muscle hypertrophy in this post-mitotic tissue (Lepper et al. 2011; McCarthy et al. 2011; Murphy et al. 2011; Relaix and Zammit 2012; Sambasivan et al. 2011; Schiaffino et al. 1976). Furthermore, much information has been gleaned about the mechanisms driving skeletal muscle cell development, repair and regeneration through the study of isolated and cultured muscle cells of satellite cell origin in vitro where they can divide, differentiate and be subject to both experimental manipulation and detailed biochemical and molecular analyses.

Although the very early work was conducted in primary cell populations, challenges occurred with mixed cell types in primary skeletal muscle and a major challenge to primary cell culture experimentation is the limited replicative capacity of primary cells first characterised by Hayflick and Moorhead and termed replicative senescence (Hayflick and Moorhead 1961). To overcome the issues of replicative senescence, immortalised cell lines of skeletal muscle origin were developed providing a means to study muscle cell physiology without confounding factors present in whole-body systems (Cornall et al. 2012).

### Cell lines

The advantages of the immortalised approach lie in the limitless supply of identical cells, which reduce the need for sacrifice of further animals and allow reproducibility of experiments between laboratories. Richler and Yaffe (1970) cultivated the L6 line from rats, Yaffe & Saxel (1977) developed the C2 murine cell lines through selective, serial passage of myoblasts cultured from C3H mice after a crush injury. Blau et al. (1983) subsequently created an immortalised ‘sub clone’ of C2’s, termed the C2C12 cell line which since have become the most widely used muscle cell line. Immortalized human skeletal muscle cell lines have also more recently emerged as tools for studying neuromuscular disorders and muscle cell function (Mamchaoui et al. 2011; Pantic et al. 2016). Importantly, both healthy and diseased immortalized myoblasts preserve the genetic and myogenic expression characteristics of their parent primary populations (Thorley et al. 2016). Thus, they are valuable when trying to understand the disease phenotype and when investigating the potential for new therapeutics when repeated sampling of primary cells is rightly considered unethical.

There are however a number of limitations to using immortalised cell lines. These include an increase in possible genetic abnormalities due to long-term culture, reduced physiological relevance, reduced cell population heterogeneity and altered gene expression and cellular processes (Voloshin et al. 2023). Immortalised cells are also the progeny of a single cell and therefore do not reflect the biological heterogeneity in responses to the same stimulus of different cells from different individuals. Any experiments undertaken using immortalised cell lines are technical replicates of a biological replicate of *n* = 1 (Bell 2016). Utilising multiple different cell lines of the same condition are needed to show efficiency of treatment across different genetic environments.

The expanding use of induced pluripotent stem cells is an interesting avenue as it opens the possibility for generating myogenic cell lines from less invasive procedures than muscle biopsies which retain the donor genetic phenotype. Thus, there is an opportunity to increase cell bank diversity to explore rare disease populations and more vulnerable patient populations. Additionally, the advances in genetic editing techniques, such as CRISP-Cas9, are further expanding the possibility for highly characterised genetic diseases where it is possible to manipulate immortalised non diseased cell lines to express the disease phenotype.

### Human primary cells

Although there are increased complexities, variability and challenges in sample collection, the study of primary cells offers greatest physiological relevance. Understanding human muscle ideally requires the use of human models wherever possible and there is a strong case for the use of human primary cells across the research spectrum from fundamental muscle biology through to translational research where the interindividual variability of humans and environmental influences are of interest to study. As we have mentioned, there are cases where the most appropriate model may be an immortalised cell population (e.g. some disease states). However, even here human primary cells have still been used shedding much light into these diseases and helping develop potential therapeutics (Massenet et al. 2024). This review will not go into detail of disease models, and they have been reviewed elsewhere (Bombieri et al. 2024; Wang et al. 2019).

### Methodological considerations of human primary muscle cell culture

When deciding to use human primary cells as the model system there are methodological considerations for the acquisition of this material which can be both ethical and practical. Like most human research to date, there is sparse literature investigating sexual dimorphism in muscle regeneration, one study suggests that females have a blunted myogenic cellular response to eccentric exercise compared to males (Fortino et al. 2022). However, to our knowledge there is currently no literature specifically investigating sex differences in myogenic cell function in vitro.

There are over 600 different muscles in the human body, and myogenic cells may be variable in a muscle-dependent manner; for example, relatively little is yet known about muscles of the head (e.g. extraocular, masseter, laryngeal), diaphragm and trunk, compared with limb muscles (Randolph and Pavlath 2015). Additionally, each muscle has a different composition of muscle fibre types which can lead to strikingly different properties of satellite cells derived from predominantly slow-twitch or fast-twitch myofibres (Lloyd et al. 2023).

From an ethical perspective, muscle tissue can most readily be obtained when being sampled for diagnostic or surgical purposes, such as discarded material obtained during hip and knee replacement. It is important to state from where, and how, the surgical samples were obtained because they may not represent a healthy physiological condition for muscle due to systemic effects of the medical condition, medication or preoperative requirements which could affect the biology of the samples. Alternatively, and commonly, muscle tissue can be obtained from volunteers in research studies using muscle biopsy samples obtained using the Bergstrom needle (Bergström and Hultman 1967; Evans et al. 1982) or conchatome (Baczynska et al. 2016) techniques. This is usually from the vastus lateralis muscle of the quadriceps. The muscle of choice, the anatomical site for sampling with respect to tendon proximity and depth of sample will all affect the composition of muscle sampled and potentially translate into in vitro experiments (Bechshøft et al. 2019a, b; Williams et al. 2022). What is evident is that in most cases viable cells can be grown from any amount of tissue, however most studies report using 100–200 mg of starting tissue. Investigating the behaviour of immediately isolated satellite cells warrants further investigation to determine which in vivo features maybe be initially retained in culture but subsequently lost as the population expands (Barruet et al. 2020; Charville et al. 2015). An important additional note, what is widely observed amongst groups who regularly work with human primary cells, is that in some instances biopsies do not yield viable populations, and it is not known why these rare incidences occur.

Interestingly, very limited attention is paid to the human source of the primary cells and there are now commercially available human primary muscle cells. They are of variable origin, from unknown participants, often from waste material during surgical procedures and vary in desmin purity. Due to the interindividual variability between humans it is critically important to address this within the design of the experiment. Owens et al. (2013) compared 4 commercially available primary cells populations and saw difference both between the individuals but also from the culture conditions recommended by the companies including being unable to expand one of the populations. Most work on human primary cells has not paid sufficient attention to the source of the cells which will be a significant contributing factor to the variability and often conflicting results observed.

### Isolation and sorting of primary human muscle-derived cells

Once a sample of muscle tissue has been obtained there are numerous protocols which have been developed for the isolation of primary cells. These fall into two main approaches - explant or enzymatic digestion. Explant, where the tissue is left in culture and cells migrate out is suggested to be the more biologically relevant (Decary et al. 1996). A quicker approach, but one which is potentially more stressful and damaging for the cells, is to mechanically mince the tissue in combination with enzymatic digestion using a combination of collagenases and dispases, trypsin etc. to break down the extracellular matrix and free the satellite cells and other mononuclear cells from their environments (George et al. 2010). There have been multiple method papers published outlining the basic principles and specificities of different protocols but there now seems to be a convergence around enzymatic digestion of mechanically minced tissue to release the mononucleated cells from the extracellular matrix as being the isolation method of choice, reviewed in (Romagnoli et al. 2021).

A challenge of working with skeletal muscle is that, compared to other tissue types, it yields low numbers of mononucleated cells per mg of tissue. Therefore, most cell culture protocols require periods of cell population expansion prior to experimentation to produce sufficient cells. An important note is that it is not customary to check for cells not extracted from the tissue. Since this process is largely dependent on enzymatic, and to some extent mechanical, breakdown of the extracellular matrix (ECM), it is possible that age and disease states where ECM accumulates may lead to a lower yield of cells, or a selective retainment of specific subpopulations that could influence the outcome. Although the immediately isolated mononucleated cell population is highly heterogeneous (Rubenstein et al. 2020), the culture media and conditions favour the attachment and cell cycle entry of satellite cell derived desmin positive muscle precursor cells and fibroblastic cells expressing TE7, which start to dominate (Agley et al. 2015). Thus, human primary cell studies have been confounded by variability of these two cell populations within the initial cell populations. This issue is compounded by the fact that information on myogenic purity (e.g. % desmin positive cells) of cultures used in experiments is often missing from published studies.

To address the issue of mixed cell populations in culture, different cell sorting methods can be employed. Beginning with simple pre-plating approaches exploiting the quicker attachment time of fibroblasts to enrich myogenic cell percentages (Blau and Webster 1981), these have subsequently evolved to using cell-specific markers for sorting. The main challenge here being that the prime marker for satellite cell identification (Pax7) is not a cell surface, but a nuclear marker. This makes it problematic as a sorting marker for primary human cells because it requires either genetic labelling of the protein or permeabilization of the cell population. Furthermore, Pax7 is also lost over time in culture as the cells become more committed myoblasts (Boldrin et al. 2010). Fortunately, other surface markers began to be discovered such as CD56, also known as NCAM, which is expressed in human, but not murine satellite cells (Boldrin et al. 2010). Additional cell surface markers CD82 and CD318 have subsequently been identified via surface protein profiling (Uezumi et al. 2016).

Exploiting cell surface markers, Fluorescence Activated Cell Sorting (FACS) has been used to sort for myogenic cells by positively selecting for CD56^+^/CD29^+^ cells (Xu et al. 2015) or negatively selecting for CD34^−^/CD31^−^/CD45^−^ cells (Charville et al. 2015). Both sorting strategies yielded cell populations with very high percentages of desmin^+^, myogenic cells. Whilst FACS is a highly accurate sorting method, it requires cells for positive and negative controls for the initial experimental set up, which can accumulate especially when using multiple markers. Magnetic activated cell sorting (MACS) has been shown to be much gentler on the cells when compared to FACS as well as being a lot easier and quicker to perform in a laboratory setting (Sutermaster and Darling 2019). A CD56^+^ MACS sorting protocol has been developed which has been stringently tested to show that CD56^+^ cell sorts produce a > 90% desmin^+^ population in human myogenic cells (Agley et al. 2013, 2015). Although CD56 is a good myogenic sorting marker at early passage we and others have shown that the number of CD56^+^ cells declines over time in myogenic cultures and a higher number of desmin^+^ cells appear in CD56^−^ sort fractions (Francis et al. 2022; Massenet et al. 2024). The optimal sorting protocol will be dependent on the research question. Where MACS would be better suited to single positive marker sorting on expanded populations and FACS better for multiple markers sorting using combinations of positive and negative marker expression to select rarer cell types and on unexpanded cell populations.

### Transition of States of myogenic cells in culture

**Fig. 1:**
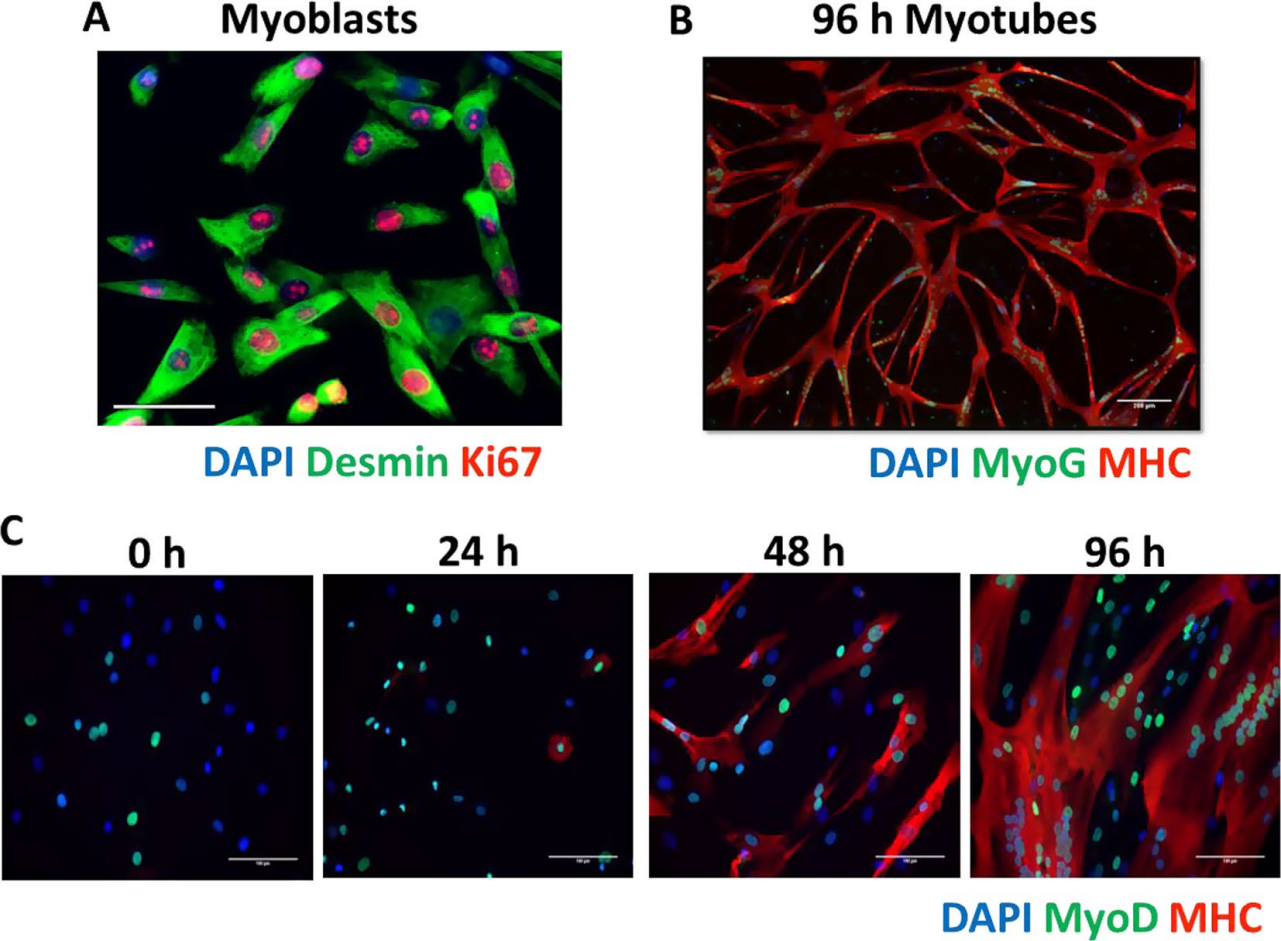
States of human myogenic cells under culture conditions. (**A**) Proliferating Desmin^+^ myoblasts expressing cell cycle progression marker Ki67 after CD56^+^ magnetic bead sorting. (**B**) Representative image of myosin heavy chain expressing myotubes formed from myoblasts after 96 h of differentiation. The majority of fused nuclei are also expression myogenic regulatory factor myogenin. (**C**) Time course of differentiating myoblasts into myotubes images taken every 24 h over 96 h. Images show the increasing number of nuclei expressing myogenic regulatory factor MyoD and the onset of myosin heavy chain expression

The research question being answered will determine whether myogenic cells need to be studied in a proliferating, differentiating or differentiated state (Fig. 1). An important question to address early on is whether the phenotype to be investigated is retained by cells under culture conditions? For example, genetic differences such as mutations in dystrophin proteins will be retained in culture. However, the disease phenotype may not be observed until higher order structures such as myotubes or once more mature sarcomeric machinery has been developed. Also of importance is that any retained phenotype observed in myotubes must have been experienced and etched into the “memory” of satellite cells as the myotubes studied have differentiated from satellite cells removed from the in vivo environment causing the phenotype.

During the tissue digestion process the muscle repair and regeneration pathways have already been activated, awakening satellite cells from quiescence into an active proliferative state (Machado et al. 2017). Under culture conditions they quickly become myogenic precursor cells losing satellite cell markers such as Pax7 and increasing early myogenic factors as they commit to the myogenic lineage (Boldrin et al. 2010). In vivo these “myogenic precursors” or “myoblasts” are a transient population dividing rapidly before exiting the cell cycle to predominantly fuse with muscle fibres and their nuclei become terminally differentiated myonuclei, while some cells re-enter quiescence and return to the niche as satellite cells (Gudagudi et al. 2020). Investigating activated satellite cell function and behaviour has been utilised in studies relating to ageing and disease where the ability of satellite cells to divide and differentiate could be affected or limited.

Myoblasts undergo differentiation *in vivo;* therefore, a more functional assessment of behaviour is to monitor this ability. In culture, human myoblasts can be induced to differentiate by allowing then to become confluent in high serum conditions, as well as at lower confluency in low serum conditions. The myogenic differentiation assay is therefore a model system of the early stages of regeneration and is useful for identifying any functional differences in vitro which may reflect the in vivo phenotype. The time course of the myogenic regulatory factors cascade can be monitored to determine if condition or treatment has had an impact on the differentiation of myoblasts, for example differentiation of human cells can be blocked by incubation with TGF-β (Alsharidah et al. 2013). Using cell culture, Wnt signalling has been shown to be essential for myogenic differentiation, but the mechanism is subtly different in human cells compared to mice (Agley et al. 2017).

Once differentiated, the myotubes formed can also be investigated as a potentially more mature, muscle fibre-like, structure. The functionality or response to treatment can be manipulated and investigated. However, human primary myotubes formed in culture express embryonic MYH3 and neonatal MYH8 myosin heavy chains (MHC) suggesting they are not truly representative of adult fibre types (Schiaffino et al. 2015). Lund et al. (2018a, b) did not observe any correlation between MHC expression in vivo muscles and differentiated myotubes, demonstrating that myotubes differ from donor muscle with respect to MHC expression and that the differentiation of satellite cells to specific muscle fibre types is not predetermined (Lund et al. 2018a, b). Further functional assays that look at regeneration of formed myotubes include the scratch assay, where wells containing myotubes are “scratched” with an implement that damages the myotubes, and the time taken to regenerate the damaged myotubes is recorded. More advanced, bioengineered culture systems have been developed and are briefly discussed in the future work section of this review.

### Use cases for human primary muscle cells

The following sections outline how and where human primary muscle cell culture has been used to investigate different biological questions. We focus on studies utilising human primary cells as the main model, with sufficient numbers of biological replicates.

### Ageing and senescence

There is an inherent loss of skeletal muscle mass with age even in those who maintain high levels of physical activity (Harridge and Lazarus 2017). Histological evidence supports a selective atrophy of type II muscle fibres contributing to an overall slower phenotype which could also be driven by selective loss of type II muscle fibres or Type II motor unit loss and neuromuscular remodelling of remaining slow motor units (Wilkinson et al. 2018). Irrespective of the mechanisms the changing profile of skeletal muscle fibre composition as well as other structural changes such as infiltration of fat and connective tissue are likely to alter the composition of extracted cell populations.

Within the immediately isolated satellite cells of mice, time-to-first cell division (TTFD), a suggested proxy for stem cell fitness, was shown to be longer in older mice ~ 60 h compared to ~ 40 h from young mice (Brett et al. 2020; Rodgers et al. 2014). However, human satellite cells from young donors have a longer, 3–4 days, TTFD compared to mice (Barruet et al. 2020; Charville et al. 2015). To our knowledge, no study has investigated age-related differences in TTFD of human satellite cells.

Replicative senescence is a problem for all human primary cell culture studies. It was hypothesised that cells from older people would have undergone more divisions during life and would therefore have a reduced proliferative capacity. Early studies passaged explanted myogenic precursor cells from individual muscle biopsies across the whole human life span, from 5-day old through to 86 years old, until they reached proliferative arrest (Decary et al. 1996, 1997; Lorenzon et al. 2004a, b; Renault et al. 2000a, b; Schäfer et al. 2006). Skeletal muscle cells from a 5-day old infant could divide 55–60 times which quickly declines to roughly 15–20 population doublings in cells extracted across all adult aged donors with differences mainly between different laboratory groups. Later studies employing larger grouped sample sizes have also shown that the proliferative capacity of adult human stem cells in culture was unaffected by donor age (Alsharidah et al. 2013; Barberi et al. 2013; Bigot et al. 2015; Pietrangelo et al. 2009). This agrees with in vivo studies in ageing humans where experimentally induce necrosis and subsequent muscle regeneration demonstrated the excellent capacity for muscle regeneration in older men aged 60–73 year (Karlsen et al. 2020).

**Fig. 2:**
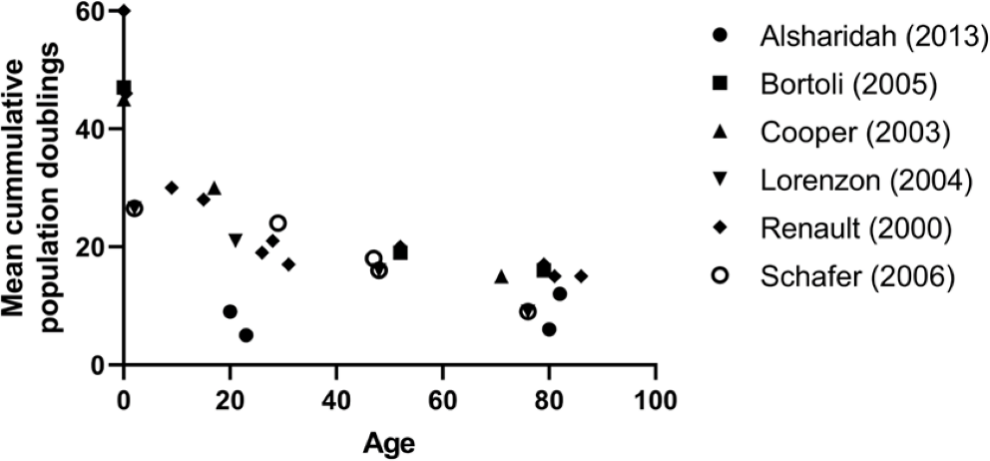
Population doubling limits of highly myogenic skeletal muscle cell populations. Cumulative population doublings for desmin positive cell populations from all published studies that reported desmin purity and population doubling numbers. Only samples which where at least 80% desmin at senescence were included here. Cell populations from individual studies are represented by different symbols

Most studies have low *n* numbers due to the labour-intensive nature of the experiments with some populations requiring over 100 days of continuous culture to reach replicative senescence (Barberi et al. 2013). Figure 2 depicts the data from all published studies where age of donor, number of mean population doublings and myogenic purity have been reported and plotted for mean population doublings against age of donor. The decline in proliferative capacity from birth to adulthood is striking compared to the relative stability during the adult years. There are high activity phases for satellite cells during development and through adolescence as skeletal muscle develops and grows. Once into adulthood, proliferative capacity of myoblasts is relatively stable with no obvious trend for lower proliferative capacity in older adults compared to young adults although it is noteworthy that there is a large degree of individual variability. Further to this, muscle cells extracted from a cadaver, aged 95 years at death and sampled 17 days after death, were still able to provide division and fusion competent myoblasts (Latil et al. 2012), highlighting the robust and resilient nature of the satellite cells as they reside in a quiescent state in their niche.

In contrast to animal data, the total proliferative capacity of human myogenic cells is similar across the adult samples. Some studies reported that cells from older adult donors took longer to grow out of their explants, had a lower percent desmin positive cells in initially expanded cultures and had a higher percentage of non or slow dividing cells, as measured by BrdU incorporation, suggesting some internal differences persisting in these cells post isolation (Barberi et al. 2013; Bigot et al. 2015; Decary et al. 1997). Furthermore, studies showed a down-regulation of skeletal muscle structural protein desmin over time as the cells approached senescence, as well as an increase in the number of desmin negative cells. Francis et al. (2022) closely monitored population purity in highly enriched desmin^+^ cell populations and saw that even highly myogenic cultures can become overrun with fibroblasts with serial passaging.

It is important to consider that studies using in vitro replicative capacity as a measure of ageing are likely to misrepresent the in vivo cell population because any senescent cells extracted will be unable to proliferate and will therefore be rapidly outgrown by the proliferation of healthy, functioning cells. The proliferative capacity of cell populations is also not a very sensitive measure because population doublings are exponential and therefore the number of cells after 20 PDT is 100’s of millions of cells, making any slight differences hard to observe.

Overall, under culture conditions there is a significant proliferative capacity from myogenic cells isolated from humans across the lifespan, however they must also be functionally able to differentiate if they are to repair damaged tissue. The early studies, with few donors, also looked at differentiation capacity from their different aged human donors.Renault et al., (2000a, b) showed that myotube formation, as measured by fusion index, was similar between different aged adults. In contrast, Lorenzon et al. (2004a, b) andJacquemin et al., (2004) did show age related impairment in fusion capacity, with cells from a middle aged donor taking longer to fuse but eventually formed myotubes whereas cells from elderly donors were unable to form myotubes even after 10 days. However, as both studies only used one biopsy per age, limited conclusions can be drawn from their findings.

Studies using larger population sizes show conflicting results. Samples from 3 young (29–48 years) and 6 old (69–87 years) donors showed a trend for poorer fusion of myoblasts from older (33.7%) compared to younger (56.7%) adults (Fulle et al. 2005). However, the conclusions are limited by low desmin purity, around 60%, in both young and old adults. A similar study by Beccafico et al. (2007), also using mixed cell populations from 3 young (~ 30 years, ~ 55% desmin) and 3 old (~ 83 years, ~ 40% desmin), showed a lower fusion index from old donor cells, ~ 25%, after 7 days of differentiation than young donor cells, ~ 45%. The first study of differentiation capacity with sizeable groups of participants (*n* = 21) was performed by(Beccafico et al. 2011) who showed that old donor cells differentiated less efficiently than those from young donors and had lower Pax7 expression, reduced ability to express MyoD, Myogenin and MHC, as well as lower RAGE and higher S100B levels.Sousa-Victor et al. (2014) also showed lower fusion from older donors but they only measured fusion after 48 h, whereas(Beccafico et al. 2011) took their differentiation assay out to six days. Similarly, Bigot et al. (2015) showed increased fusion of older donor cells, suggesting that there were fewer unfused cells due to epigenetic repression of SPRTY-1 expression preventing the cells‘ ability to return to quiescence. This would leave differences in the reserve cell populations rather than the differentiating populations and could suggest a possible mechanism for the reduced satellite cell number with ageing.Bechshøft et al. (2019a, b) saw no different in differentiation index but did see an age-related reduction in fusion index. In contrast, Alsharidah et al. (2013) showed no difference in fusion index, myotube size or expression patterns of MyoD, myogenin or MHC between young and older donors.

Overall, the fusion competence of cells from different aged humans is variable with heterogeneity between studies and between donor cells, even when desmin purity is controlled for. The main conclusion to draw from these experiments is that every study looking at muscle cells from aged biopsy donors have been able to grow viable cell populations. This would suggest that even in the oldest old, and even post death, there is a remnant satellite cell population capable of expanding and undergoing differentiation under good environmental conditions.

Even though the data supporting in vivo phenotype retention is conflicted, when human myogenic cells are aged under culture conditions their growth and function is altered with cells taking longer to divide, accumulating damaged and producing fewer and smaller myotubes (Alsharidah et al. 2013; Francis et al. 2022). Whether this cell culture induced senescent phenotype is representative of any features of in vivo ageing muscle is still not understood. For example, the characterisation of senescent populations of skeletal muscle cells in vitro could provide cell type specific markers of senescent cells which could be used for determining the senescent state of in vivo populations of cells or model aspects of muscle ageing such as anabolic resistance. Yet what is most apparent from these studies on different aged donors is that future studies need to consider from whom the samples have been obtained. Older age is highly heterogeneous, with the older phenotypes varying markedly in physiological function. Physical activity is key to obtaining the appropriate phenotype for any given age, as inactivity is deleterious to muscle structure and function (Harridge and Lazarus 2017).

### Physical activity effects on human primary skeletal muscle

One predominant consideration to human muscle is physical activity and exercise. Muscle is exquisitely sensitive to mechanical and metabolic signals. An interesting comparison is that the gas concentration and nutrient availability in cell culture conditions may be an in vitro analogue to the superior circulatory environment seen in physically active older adults. Physically active older people have been shown to have a muscle morphology more similar to young people than age matched sedentary people (Mikkelsen et al. 2017; Mosole et al. 2014; Sonjak et al. 2019). A key area of skeletal muscle ageing that has yet to be fully investigated in cell culture studies is whether the effect of exercise is retained in vitro. This can partly be attributed to a scarcity of studies, as well as the use of vastly different modalities of exercise. Barberi et al. (2013) showed that the proliferative capacity of cultured myoblasts was similar between young, old active and old sedentary individuals (Barberi et al. 2013). Similarly, both Balan et al. (2020) and Soendenbroe et al. (2022) observed no effect of a physically active lifestyle on markers of cellular senescence and myogenesis, respectively, in cultured human myoblasts. However, a follow-up study by Soendenbroe et al. (2024) which assessed the ability of myoblasts and fibroblasts to protect and preserve motor neurons in vitro, observed a strong effect of exercise, both in myoblasts and fibroblasts. This opens another avenue, where it is not so much the intrinsic capabilities of the cells that are altered by prior exercise, but more their interaction with the cells surrounding them. Lastly, Bechshøft et al., showed that just a single bout of heavy resistance exercise was imprinted in isolated myoblasts when they were collected seven days later as evidenced by lower myogenic regulatory factor MyoD and senescence associated cell cycle inhibitor p16. This hints at a dynamic regulation of “cell memory” (Bechshøft et al. 2019a, b).

### Insulin sensitivity and metabolism

One key aspect of cell physiology that is conferred by exercise, and which is retained in vitro, is glucose and lipid metabolism. Both Green et al., and Lund et al., found that the metabolism of differentiated myotubes was dependent on the exercise status of the donors (Green et al. 2013; Lund et al. 2018a, b). In fact, 8 to 12 weeks of in vivo aerobic exercise altered the glucose and lipid metabolism of differentiated myotubes in vitro (Bourlier et al. 2013; Lund et al. 2017). Similarly, Nemec 2021 showed improvements in myotube lipid metabolism after 6 months of exercise training in muscle samples from idiopathic inflammatory myopathy patients. As such, a picture is emerging that it is predominately cellular metabolism which is affected by prior exercise and a phenotype retained in culture.

Insulin resistance has also been shown to be retained in cultured myoblasts from people with insulin-resistance, suggesting an underlying biochemical defect (Henry et al. 1995; Thompson et al. 1996). However, conflicting results exist, with some researchers finding no difference in insulin action and signalling between cultured cells from insulin-resistant and insulin-sensitive individuals (Krützfeldt et al. 2000). Glucose uptake measurements in differentiated myotubes can reflect insulin sensitivity (Chanon et al. 2017) and insulin resistance can be induced in cultured muscle cells from both non-diabetic and diabetic subjects through hyperinsulinemia, accompanied by increased GLUT1 but unaltered GLUT4 levels (Ciaraldi et al. 1995). Additionally, cells from diabetic individuals show increased sensitivity to hyperglycaemia-induced impairment of glucose transport (Ciaraldi et al. 1995). It is not just the responsive phenotype that is retained, myotubes grown from cells isolated from people with diabetes have an altered secretome including higher levels of IL6, IL8, IL15, TNFα and follistatin compared to non-diabetic myotubes (Ciaraldi et al. 2016). Interestingly, 3D myospheres, formed by cells in suspension on ultra-low attachment u-shaped bottom plates, showed greater lipid oxidative metabolism compared with 2D myotubes model, which oxidized more glucose and accumulated more oleic acid (Dalmao-Fernandez et al. 2023). The insulin resistance phenotype seems to be retained through detectable differences in DNA methylation which are retained in cultured human myoblasts (Burton et al. 2023). These findings highlight the complex interplay between genetic and environmental factors in the development of insulin resistance within skeletal muscle.

### Treatment of cultured cells with serum

Cell culture also offers the experimental opportunity to investigate the effects of environmental factors on behaviour i.e. to mimic the circulating environment in vivo, by treating cells with serum collected from different groups of individuals. There is extensive literature on how the use of serum or plasma, derived from various sources, when added to cell culture media, alters many aspects of a myoblast’s cellular physiology. This literature is, to some extent, fuelled by dramatic findings using the heterochronic parabiosis in rodents, whereby systemic factors have been shown to play a role in impaired function of satellite cells and tissue regeneration in vivo (Conboy et al. 2005). This raises the question: Could human serum conditioned medium serve as a human analogue to the heterochronic parabiosis model? One aspect of this relates to the use of human serum conditioned medium to reduce the use of FBS, which could potentially help minimize batch variation, and appease ethical concerns (Jochems et al. 2002). Within regenerative medicine, where a goal is to inject “functional” stem cells into live humans, but the use of serum derived from other species raises concerns about pathogenicity (Tollance et al. 2024). The use of serum and plasma as condition medium has been comprehensively reviewed elsewhere (Allen et al. 2023); the general *modus operandi* is to draw blood samples from specific groups (patients vs. controls, older vs. younger adults, etc.) or at specific states (exercisers vs. sedentary, fed vs. fasted), and then apply these samples to the chosen myogenic model system. While most published studies obtain serum/plasma from multiple donors that then represent biological variables of a given group or state, the myogenic model system is in most cases the C2C12 cell line (i.e. technical replicates of one cell). There are, as far as the authors are aware, only three studies that have isolated primary human muscles from multiple donors *and* exposed these to medium conditioned with serum from multiple donors (Catteau et al. 2020; Corrick et al. 2015; George et al. 2010).George et al. (2010) isolated myoblasts from two younger individuals, and cultured these in serum conditioned medium derived from 13 younger and 9 older individuals. Surprisingly, no effect of age of the serum donor was observed at either early or later stages of myogenesis, contrasting findings later made using C2C12 cells (Allen et al. 2021). This difference may relate either to differences in the health and physical activity status of the individuals from whom blood was donated and / or differences in the sensitivity or human primary cells compared to C2C12 cells. Nevertheless, treatment with candidate inflammatory cytokines such as TGF-β demonstrate an ability to influence primary human primary muscle cell behaviour by impairing differentiation (Alsharidah et al. 2013). Corrick et al. (2015) exposed myoblasts, isolated from vastus lateralis muscle biopsies of three healthy young men, to serum from individuals, who had sustained severe burn injuries, or matched controls. Burn injury creates a strong systemic inflammatory response which increases skeletal muscle catabolism, and the authors speculated that burn injury would impair myogenesis. In agreement with this hypothesis, the ability of myoblasts to fuse and grow was severely impaired when exposed to serum from burn victims, highlighting the importance of circulating factors for muscle growth. Lastly, Catteau et al. (2020) showed that human serum was superior to traditionally used fetal bovine serum (FBS) for different aspects of myogenesis, and that serum isolated from patients with chronic obstructive pulmonary disease, a condition associated with inflammation, lead to less myotube growth. There are, as previously mentioned, several studies that have availed of the C2C12 cell line (and other cell lines) and made interesting observations on the effect of serum conditioned medium on ageing, protein feeding and various diseases. However, until these findings have been replicated with more robust study designs using primary human cells, these findings remain speculative.

An alternative to supplementing media with serum or plasma from donors is the use of “conditioned medium”, which is collected from cultures where isolated cells have been grown. This medium contains factors secreted by the cells during culture. Importantly, many nutrients and growth factors are also taken up by the cells, explaining why the protein content of cell-conditioned medium is often lower than that of non-conditioned medium (unpublished data). Skeletal muscle in general Roca-Rivada et al. (2012), and myoblasts specifically Brzeszczyńska et al. (2018), are known sources of circulating factors that may act in auto-, endo-, and/or paracrine manners. These muscle-secreted factors have been shown to stimulate various processes in other cells. For example, as early as 1977, it was demonstrated that spinal cord derived cells (neurons, astrocytes, glial cells etc.,) benefitted from the presence of muscle cells (Giller et al. 1977). More recently, in vitro experiments using microfluidic devices (i.e. separate compartments with diffusion) have shown that myoblasts stimulate motor neuron neuritogenesis, further underscoring the importance of muscle-derived factors (Southam et al. 2013). The primary advantage of using cell-conditioned medium is that the origin of the factors in the medium can be precisely traced back to the cultured cells. The composition of the media and its effect on other cells can then be directly investigated. Crucially, the effect of secreted factors from other cell types on myoblasts can also be studied, for example tendon cells increase myotube fusion index (Tsuchiya et al. 2022), as can the impact of potentially deleterious factors such as those secreted by cancerous cells, potentially having a direct effect on cachexia (Krapf et al. 2022).

### Fibroblasts– friend or foe?

We have focused on the CD56^+^ sorted populations consisting of satellite cell-derived myoblasts; however, the sorting process also yields a CD56^−^ population consisting primarily of fibrogenic precursor cells, termed Fibro-Adipogenic Progenitors (FAPs) or simply fibroblasts (Agley et al. 2015). This population is often discarded, as they are often being viewed as a frustrating contaminant to myogenic purity of primary muscle cell culture. However, the physiological importance of fibroblasts has in recent years been revised in the context of their fundamental role in supporting satellite cells during muscle repair (Farup et al. 2021; Murphy et al. 2011).

**Fig. 3:**
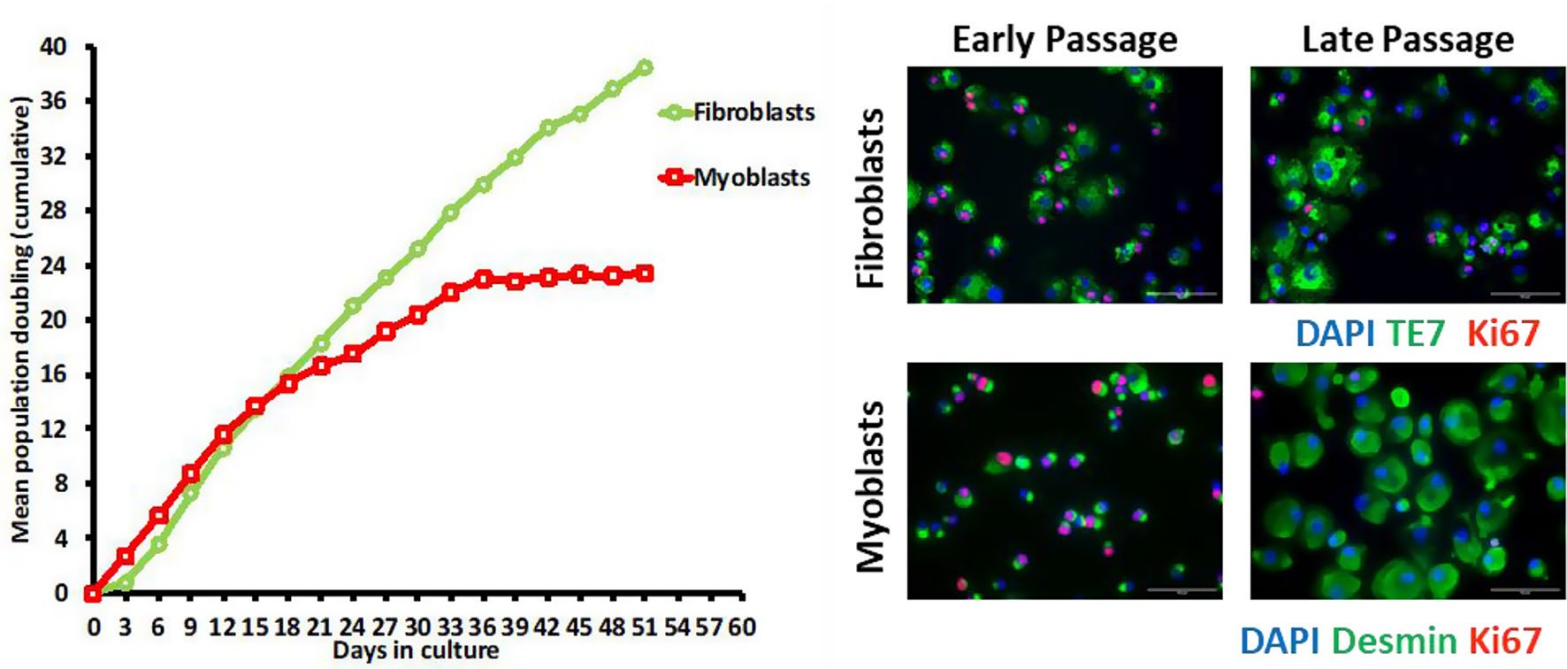
Representative population doubling time curves for myoblast and fibroblast populations extracted from the same biopsy. Population doublings recorded every 3 days for CD56 + and CD56- cell populations extracted from the same muscle biopsy. The myoblasts reach replicative senescence whilst in the same time frame fibroblastic cells seem to maintain a continued proliferative capacity. Images represent early passage and late passage cells for both TE7 positive fibroblasts and Desmin positive myoblasts. At early passage both cell types have high numbers of Ki67 expressing cell whereas at late passage myoblasts are no longer expressing Ki67 whereas similar number of fibroblasts are expressing Ki67 and are undergoing cell division. Sample was obtained from a male donor aged 25 years (REC reference 16-LO-1707)

Compared to myoblasts, fibroblasts have a much larger capacity for cell division. In 2022, Francis et al. followed the growth trajectory of highly enriched myoblasts and fibroblasts from the same muscle biopsies. Within the myoblast populations it was evident that adult myoblasts reach replicative senescence in roughly 20 population doublings. However, this was confounded by the fact that even when highly enriched for desmin^+^ cells the remaining fibroblasts can out compete and over grow the senescing myoblast populations. This was further confirmed by the replicative capacity of the fibrogenic populations maintain a high level of proliferation well beyond the myogenic replicate span (Fig. 3). The potential for cell population shifts even in highly enriched starting populations further shows the importance of assessing cell population purity, e.g. desmin purity for myogenic cells, at the start of experimentation.

Fibroblasts from human muscle have also been shown in some (Joe et al. 2010; Mackey et al. 2017; Mathew et al. 2011) but not all (Bechshøft et al. 2019a, b) studies, to positively stimulate myogenesis. On the other hand, fibroblasts can, unlike myoblasts, undergo both adipogenic or fibrogenic differentiation (Agley et al. 2013; Arrighi et al. 2015; Farup et al. 2021), and excessive fibroblasts accumulation and/or activation has been suggested as the main driver of fibrosis and adipogenic tissue accumulation observed in ageing muscle (Plikus et al. 2021). These polarized findings open the question whether all fibroblasts are equal?

From single-nucleus RNA sequencing the proportion of fibroblasts in human skeletal muscle is estimated to be approximately 10% of all nuclei (Lai et al. 2024), and enumeration of fibroblasts in human skeletal muscle by immunofluorescence on tissue sections estimates 1–2 times as many fibroblasts relative to satellite cells (Fry et al. 2017; Mackey et al. 2017). Such high abundance is itself indicative of an important physiological role, and depletion of fibroblasts has also been shown to impair regeneration following injury (Wosczyna et al. 2019). This posits the fibroblasts as a cell type that, like myoblasts, responds to various insults by increasing proliferation, and changing its gene expression. But unlike myoblasts, fibroblasts appear to serve a dual role, where they in some instances are required for the normal tissue homeostasis, and in other instances contribute to certain negative outcomes. Focused investigations into skeletal muscle origin fibroblastic cells are vastly important and a growing number of laboratories are starting to explore their heterogeneity (Contreras et al. 2021).

### Future directions

In the past 100 years there has been some success in transferring results from in vitro work into the clinic (Kohler and Milstein 1975; O’Connor 1981; Old et al. 1968; Pellegrini et al. 2009). However as relates to muscle physiology and pathology, cell culture work has yet to make this step. The number of groups utilising human primary myogenic cells is increasing with most implementing sorting strategies and at least some genetic heterogeneity in their populations. As well as improving and gaining deeper understanding of myogenic cell behaviour recent technological advances are allowing for a much deeper investigation of all cell types within skeletal muscle and creating more physiologically relevant models.

### Single cell studies

The advent of single cell and single nuclei analysis have brought into focus the heterogeneity and diversity of cell types contained within skeletal muscle and shows promise as being able to more precisely define cell phenotypes. Eleven mononuclear cell types have been identified in human muscle, including at least two fibro-adipogenic progenitor subtypes, two subtypes of endothelial cells, adipocytes, smooth muscle cells and pericytes, as well as populations of immune cells (Rubenstein et al. 2020). Muscle stem cells have been found to bifurcate into quiescent and early activated subpopulations with distinct gene expression profiles associated with aging, obesity, and impaired regeneration (De Micheli et al. 2020). Muscle myonuclei do not significantly appear in single cell preparations, presumably because they do not have intact cell membranes, but they can be found and studied using single nuclei RNAseq (Karlsen et al. 2023; Kedlian et al. 2024; Lai et al. 2024; Perez et al. 2022). Location-specific populations of myonuclei have been identified e.g. neuromuscular junction and myotendinous junction specific phenotypes and disease-specific populations in muscular dystrophies (Williams et al. 2022). These technologies are still in their infancy especially when looking at human skeletal muscle with many of the donor samples minimally characterised and often from surgical patients that may not fully reflect physiologically healthy muscle biology. Yet the possibility to investigate further specific sub populations with skeletal muscle is an avenue of research ripe for further investigation. The need to identify sub population specific markers which can be used for sorting are required. Subpopulations of satellite cells have been observed in vitro, which seem to have distinct abilities to return to quiescence and have different metabolic profiles (Bigot et al. 2015; Bouche et al. 2023). CD56^+^/CD15^+^ were suggested to have adipogenic potential, however they were found to be extremely rare, < 1% of CD56 + cells and did not undergo adipogenic differentiation (Agley et al. 2013). Combining these in vitro observations with in vivo single cell and nuclei datasets will lead to new understanding and determine if these sub populations are physiologically relevant or artifacts of culture. For example, single-cell RNA sequencing identified transcriptionally distinct subpopulations of PAX7 + satellite cells, including a CXCR4/CD29/CD56/CAV1 + subset with enhanced engraftment potential (Barruet et al. 2020).

### Muscle organoids and culture conditions

When culturing cells in vitro there is always a caveat that the cell culture environment significantly influences cell behaviour, impacting survival, proliferation, and differentiation. Factors such as medium pH, osmolarity, gas composition, and temperature fluctuations can affect cell responses (Bertoncello 2019). Traditional cell culture methods often fail to replicate physiological conditions, particularly regarding oxygen levels and three-dimensional environments (Abbas et al. 2021). Tissue level oxygenation is much lower than environmental, with extensive work showing altered behaviour and responses to treatment differed when cells were exposed to physiological oxygen tensions (reviewed in Keeley and Mann 2019). A primary example would be that standard cell culture studies often have an environment of 20.9% oxygen (normoxia) that can have adverse effects, whereas human myoblasts have been shown to proliferate more in lower oxygen at 5% and even at 2–3% O_2_ which better resembles their physiological condition at the tissue level (Martin et al. 2009; Sellathurai et al. 2016).

Culture systems which mitigate some of these physiological differences may lead to a more complete identification of the products secreted into the medium as cells grow and differentiate. Linked sequential perfusion systems in which cells that are closely related in vivo are perfused with media from one cell type stimulated with a chemical specific to that cell and then culture perfusate is allowed to percolate over all the succeeding cell types could shed light on how these cell types are interacting in vivo (Edington et al. 2018).

While significant progress has been made, challenges remain in achieving fully mature and functional muscle tissues in vitro. Different substrates, polystyrene dishes and collagen-coated surfaces have also been shown to alter cell cycle progression and biochemical profiles (Gargotti et al. 2018). Fibroblasts are a key progenitor cell and the main source of extracellular matrix production. Use of different ECMs can cause more rapid cell organisation and differentiation of myogenic cells than single matrix glycoprotein substrates, which are still present in serum free media, potentially representing an alternative approach to the use of serum (Chaturvedi et al. 2015). Clearly the ECM is more important than simply providing structural support for the myofibre, and along with the interaction between satellite cell cilia and ECM in mechanosensing and signalling to the nucleus, warrants further investigation in vivo and in vitro (Ng et al. 2021).

More recently, techniques using three dimensional cultures have been suggested as a potential way forward for the discovery of new pharmaceuticals (Breslin and O’Driscoll 2013). Various cell culture methods, well plates, microfluidics, organoids, and bioprinting have been employed to create sophisticated skeletal muscle tissue models which have been touted to have potential for drug discovery, disease modelling, and personalised medicine applications because these engineered tissues better replicate muscle development, genetic diseases, and regeneration processes (Khodabukus et al. 2018; Moyle et al. 2020).

These advanced substrates and matrices enable more accurate exploration of cell-matrix interactions and cellular responses to temporal variations in their environment, potentially improving the translatability of in vitro studies to in vivo conditions (Abbas et al. 2021; Kim and Hayward 2012). A further interesting difference between human and mouse myoblasts is that human myoblasts are able to invade a collagen gel due to collagen 14 expression whereas mouse myoblasts that do not express collagen 14 are unable to migrate through the gel (Lund et al. 2014). Further showcasing that as model systems advance more species differences are likely to be revealed emphasising the need to study human cells for the most direct relevance to human muscle.

There are many model systems developing concurrently which are reviewed elsewhere (Moyle et al. 2020). With skeletal muscle being an electrically activated tissue in vivo, cell culture systems have used acute high-frequency or chronic low-frequency electrical stimulation of human myotubes to mimic the effects of exercise in vitroNikolić et al., (2012). These “exercise-in-a-dish” experiments have been taken further into 3D scaffolds. For example, Nagashima et al. (2020) show that immortalised myogenic cells formed into “tissue” on their microfabricated device. The resulting tissues could generate a detectable tetanic force in response to the electrical pulse stimulation. Similarly, chronically stimulating “micro muscles” to mimic exercise can induce a hypertrophic and metabolic responses (Mills et al. 2019). Taking models even further some groups have begun to incorporate multiple cell types and supporting structures like neuromuscular junctions and vasculature (Moyle et al. 2020; Pinton et al. 2023). Using 3D spheroids of iPSC derived motor neurons applied to 2D and 3D primary human skeletal myotubes cocultured with fibroblastsRimington et al., (2021) showed they could change time to peak twitch and half relaxation times in innervated motor units suggesting the model represented physiological excitation contraction coupling. The work to develop models which incorporate human primary cells is ongoing and opens many exciting avenues for future research into muscle biology.

**Fig. 4:**
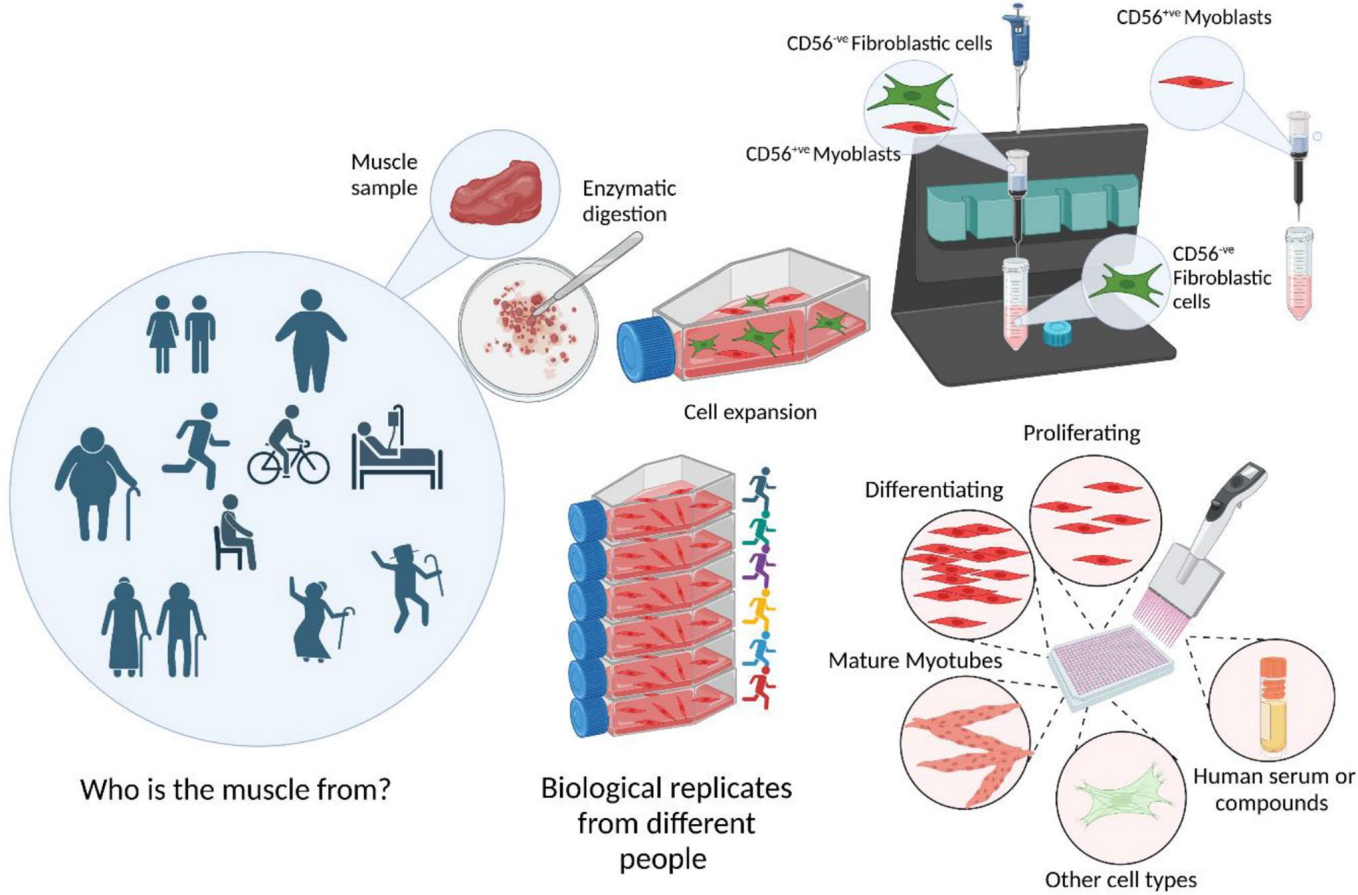
Schematic overview of important considerations for human primary cell culture. The first important consideration is the donor of the cells. Age, sex, physical activity level, disease status, previous acute exercise are all possible confounders so the more known and reported about the donor is important. Muscle sampled, location of sample within the muscle and procedure used are also considerations. Extracting cells from tissue can occur by different methods, predominantly enzymatic digestion which may not release all mononucleated cells. Purification of cell types is required from digested skeletal muscle with cell surface marker CD56 most common for enriching myogenic precursor cells. Purified myogenic cells can then be studied in different states of proliferation, differentiation or as mature myotubes in 2D or 3D cultures system with and without co culture with other cell types. Treatments can be applied such as small molecules or human serum. There is also the possibility to enrich populations of other cell types from skeletal muscle to investigate their behaviour under culture conditions. Finally, and as important as knowing the donor, is the need for biological replicates using cells from different donors to draw biologically relevant conclusions

## Conclusion

Skeletal muscle has an amazing regenerative capacity that has been known and investigated for centuries. Incredibly the regenerative capacity has also been shown to remain, at least temporarily, after death of the host. Primary cells were the original method for investigation but fell out of favour due to the challenges and limitations outlined in this review in favour of more easily maintained immortalised cell lines. However, there is a growing resurgence in human primary cell culture and when coupled with technological advances are starting to be able to answer more physiologically relevant questions which immortalised cells cannot. We summarise our approach to human primary cell culture in Fig. 4. Embracing the genetic variability and cellular heterogeneity between humans whilst being aware of the technical challenges and pitfalls of primary culture adds another layer of understanding to the fundamental biology of human skeletal muscle but could also offer so much to the translational pathways into clinical populations and societal challenges such as ageing and diabetes. What is of paramount importance when using primary human cells is knowing the origin of the cells being studied because the host’s phenotype is imprinted within the cells, and the need to use biological replicates to account for variation between individuals.

## Data Availability

No datasets were generated or analysed during the current study.
